# Alopecia areata-like patchy non-scarring alopecia as an initial presentation of systemic lupus erythematosus

**DOI:** 10.1093/rap/rkad004

**Published:** 2023-01-16

**Authors:** Yoshitaka Ueda, Takahiro Mizuta, Satoru Murata, Naoto Yokogawa

**Affiliations:** Department of Rheumatic Diseases, Tokyo Metropolitan Tama Medical Center, Tokyo, Japan; Department of Dermatology, Tokyo Metropolitan Tama Medical Center, Tokyo, Japan; Murata Dermatology Clinic, Tokyo, Japan; Department of Rheumatic Diseases, Tokyo Metropolitan Tama Medical Center, Tokyo, Japan

Key message

- Initial presentation of SLE can be patchy non-scarring alopecia.

Dear Editor, SLE is a chronic autoimmune disease with various manifestations. Hair loss is a common complaint, and can reflect SLE disease activity [1]. Diagnosing SLE in cases with alopecia as the main complaint is very difficult, because various types of hair loss are observed in SLE and there are lots of aetiologies for hair loss apart from SLE. SLE-related alopecia is classified as lupus erythematosus (LE) specific or LE non-specific. LE-specific hair loss patterns include lupus hair or DLE, whereas LE-non-specific hair loss patterns include non-scarring alopecia or telogen effluvium [2]. Non-scarring alopecia was added to the 2012 SLICC Group criteria and the 2019 EULAR/ACR criteria for SLE [3, 4], and hair loss is now considered an important clue to the diagnosis of SLE. In addition, a significant positive association has been observed between alopecia areata and SLE [5]. The hair loss pattern observed in that case was patchy non-scarring alopecia, and it is necessary to differentiate non-scarring alopecia caused by SLE from simple alopecia areata or alopecia areata associated with SLE. Here, we reported a case of SLE presenting with patchy non-scarring alopecia.

A 22-year-old female patient presented to a dermatology clinic complaining of patchy hair loss with a duration of 2 years. After ANA were found, she was referred to our department. The only other complaint besides hair loss was slight arthralgia on presentation. She appeared to be in good health and denied fever, chest pain, abdominal pain, diarrhoea, erythematous rash, RP, photosensitivity and oral ulceration. She had no medical or family history of CTD. An examination revealed patches of non-scarring alopecia on the temporal and parietal scalp (Fig. 1A). No erythema, scaling or tenderness was observed. The hair loss was confined to the scalp, and there was no evidence of malar rash, oral ulcers, lymph node enlargement or joint swelling. Dermoscopy found no characteristic features of alopecia areata, such as black dots, exclamation-mark hairs or broken hairs (Fig. 1B). Laboratory tests were positive for ANA (1:320, speckled immunofluorescent pattern) and elevated anti-DNA antibody (70 IU/ml; reference value: 0–6 IU/ml). Additionally, hypocomplementaemia with low C3 (67 mg/dl; reference value, 73–138 mg/dl) and C4 (7.4 mg/dl; reference value, 11–31 mg/dl) and a normal complete blood cell count were found. Serum free thyroxine and thyroid-stimulating hormone concentrations were within the normal range. Spot urinalysis detected no haematuria, proteinuria or red blood cell casts suggestive of nephritis. No skin diseases or any internal disease was able to explain the hair loss, and patchy, non-scarring alopecia caused by SLE was finally diagnosed based on the 2012 SLICC criteria or the 2019 EULAR/ACR criteria. HCQ was initiated. One year after the start of treatment, hair growth was observed (Fig. 1C), the anti-DNA antibody level was improved and the C3 and C4 levels were normalized.

**Figure 1.**
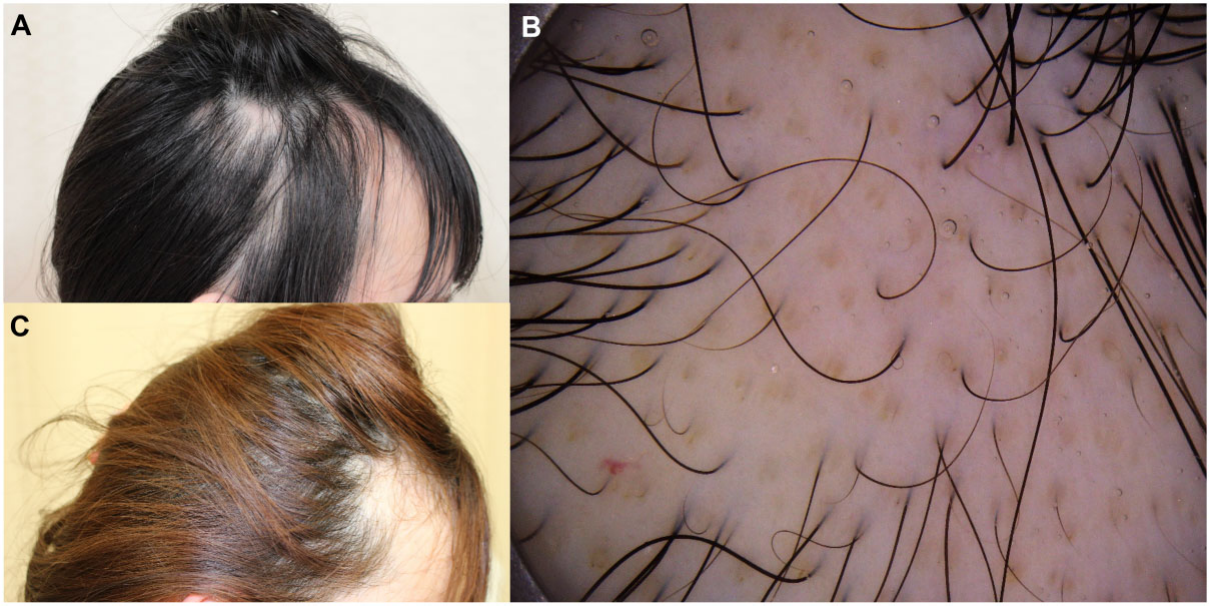
Clinical images of non-scarring alopecia before and after 1 year of treatment. (**A**) Several patches of non-scarring alopecia were observed bilaterally on the temporal and parietal scalp. (**B**) Dermoscopy revealed no black dots, exclamation-mark hairs or broken hairs. (**C**) Hair loss improved at ∼1 year after the start of treatment

In the present case study, the patient’s chief complaint was hair loss, and her arthralgia was only minor. To date there are very few reports of similar cases [6]. Alopecia is a common, cutaneous manifestation of SLE, which should be suspected whenever arthritis, malar rush, photosensitivity or oral ulceration is present, as outlined in the 2012 SLICC criteria. However, SLE is sometimes difficult to diagnose if the main, initial presentation is alopecia. SLE-related alopecia is classified as LE specific or LE non-specific, including patchy non-scarring alopecia [2]. As in the present case, patients with patchy non-scarring alopecia are usually asymptomatic, with no macroscopic changes in skin pigmentation. Therefore, the condition is often misdiagnosed as alopecia areata, a far more common disease. Besides, a significant positive association has been observed between alopecia areata and SLE [5]. In the present case, therefore, we had to differentiate non-scarring alopecia caused by SLE from simple alopecia areata or alopecia areata associated with SLE. The differential diagnosis of alopecia areata and LE-related non scarring alopecia is crucial, because it can delay appropriate treatment or lead to the use of ultraviolet light therapy, which is detrimental to patients with SLE. Gilliam *et al.* [7] reported that more than half of SLE patients with hair loss presented with non-scarring patchy alopecia, which was initially mistaken for alopecia areata. In their study of 21 SLE patients with non-scarring patchy alopecia and 21 alopecia areata patients with multiple patches of alopecia, Ye *et al.* [8] reported that in all the SLE patients, dermoscopy failed to find the characteristic features of alopecia areata, such as black dots or exclamation-mark hairs and broken hairs. These features were observed in more than half of the patients with alopecia areata, and their study suggested that scalp dermoscopy was very useful for differentiating SLE-related non-scarring alopecia from alopecia areata.

The initial presentation of SLE can be patchy non-scarring alopecia that looks like alopecia areata, and distinguishing non-scarring alopecia caused by SLE from alopecia areata requires a detailed physical and dermoscopic examination by dermatologists.

## Data Availability

Derived data supporting the findings of this case report are available from the corresponding author on request.
